# Supplementary material for: The Cost-Effectiveness of Expanding the UK Newborn Bloodspot Screening Programme to Include Five Additional Inborn Errors of Metabolism
Source: Int J Neonatal Screen. 2020 Nov 20;6(4):93. doi: 10.3390/ijns6040093 (PMC7711627; doi:10.3390/ijns6040093)
Supplement: Supplementary file 1 [file IJNS-06-00093-s001.pdf]

Supplementary material: Costs

Table S1: Diagnosis resource use

**Cost of 1st appointment**	GA1	HCU	IVA	LCHADD	MSUD
Staff	90 mins Consultant paediatrician<br>90 mins Specialist nurse<br>90 mins Dietician	60 mins Consultant paediatrician<br>60 mins Specialist nurse<br>60 mins Dietician	90 mins Consultant paediatrician<br>90 mins Specialist nurse<br>90 mins Dietician	60 mins Consultant paediatrician<br>60 mins Specialist nurse<br>60 mins Dietician	60 mins Consultant paediatrician<br>60 mins Specialist nurse<br>60 mins Dietician
Bloods	Urinary/organic acids<br>Blood plasma acyl carnatine<br>DNA GA1	Plasma quantitative amino acids<br>Total homocysteine	Plasma quantitative amino acids<br>Urinary/organic acids<br>Blood plasma acyl carnatine<br>DNA IVA	Urinary/organic acids<br>Blood acylcarnites<br>Genetic confirmation	Urinary/organic acids<br>Blood plasma AA<br>DNA MSUD
Other	Carnitine 100mg/kg/day	Carnitine 100mg per day (7 days)	Carnitine 100mg/kg/day		
**Cost of 2nd appointment (Diagnosis confirmed)**	90 mins Consultant paediatrician<br>90 mins Specialist nurse<br>90 mins Dietician	30 mins Consultant paediatrician 30 mins Specialist nurse<br>30 mins Dietician	60 mins Consultant paediatrician<br>60 mins Specialist nurse<br>60 mins Dietician	30 mins Consultant paediatrician<br>30 mins Specialist nurse<br>30 mins Dietician	30 mins Consultant paediatrician<br>30 mins Specialist nurse<br>30 mins Dietician
**Cost of 2nd appointment (False positive)**	Same as above	Same as above	60 mins Specialist nurse<br>60 mins Dietician	Same as above	Same as above

mins – minutes GA1 - glutaric aciduria type 1, LCHADD - long chain hydroxyacyl CoA dehydrogenase deficiency, MSUD – Maple syrup urine, disease, IVA - isovaleric acidaemia, HCU – homocystinuria,

S2: Management Costs

The costs of longer-term management of the conditions including routine appointments, blood tests, and dietary management were estimated from the pilot study protocol, diet management advice developed for the screening pilot [1], and expert dietician input. Healthcare costs associated with managing the conditions, such as appointments, routine tests, and the costs of dietary supplements were estimated using unit costs from routine data source [2-4]

Table S2a – GA1 costs

	Diagnosis	0-1	1-6	6-15	16+ Years
Appointments	90 mins Consultant paediatrician<br>90 mins Specialist nurse<br>90 mins Dietician<br>All x2	30 mins Consultant paediatrician x5<br>30 mins Specialist nurse x5<br>60 mins Dietician x5<br>60 mins Dietician x36 (additional apps)	30 mins Consultant paediatrician x4<br>30 mins Specialist nurse x4<br>60 mins Dietician x4	20 mins Consultant paediatrician x2<br>20 mins Specialist nurse x2<br>30 mins Dietician x2	20 mins Consultant<br>20 mins Dietician
Costs	£720	£600	£548	£171	£64
Bloods	Urinary/organic acids<br>Blood plasma acyl carnatine<br>DNA GA1	FBC x5<br>Ca, Pi, LFT x5<br>Plasma amino acids including tryptophan x5<br>Free carnitinex5<br>Micronutrients	FBC x4<br>Ca, Pi, LFT x4<br>Plasma amino acids including tryptophan x4<br>Free carnitinex4<br>Micronutrients	FBC x2<br>Ca, Pi, LFT x2<br>Plasma amino acids & ammonia x2<br>Free carnitines & acyl carnitine x2<br>Micronutrients	FBC<br>Ca, Pi, LFT<br>Plasma amino acids & ammonia<br>Free carnitines & acyl carnitine<br>Micronutrients
Costs	£376	£955	£776	£394	£203
Diet		Carnitine 100mg/kg/day	Carnitine 100mg/kg/day	Carnitine 50mg/kg/day	

		GA1 anamix to give 1g of protein equivalent per kg/day<br>60 mins Dietician x36 (additional apps)	GA1 anamix to give 1g of protein equivalent per kg/day		
Dietician cost	£0	£1,224	£0	£0	£0
Supplements costs	£0	£2,592	£3,936 - £7,453 (depending on age)	£0	£0
Total costs	£1,096	£5,371	£5,260 - £8,777	£565	£267

Mins – minutes, FBC – full blood count, ca – calcium, Pi – perfusion index, LFT – liver function tests, GA1 – glutaric aciduria type 1

Table S2b – HCU costs

	Diagnosis	0-1	1-2	2-3	3-6	6-11	11+
Appointments	60 mins Consultant paediatrician<br>60 mins Specialist nurse<br>60 mins Dietician<br>+ 30 mins each for confirmation appointment	30 mins Consultant paediatrician x5<br>30 mins Specialist nurse x5<br>30 mins Dietician x5<br>30 mins Dietician x14 (additional apps)	30 mins Consultant paediatrician x4<br>30 mins Specialist nurse x4<br>60 mins Dietician x4	30 mins Consultant paediatrician x3<br>30 mins Specialist nurse x3<br>30 mins Dietician x3	30 mins Consultant paediatrician x2<br>30 mins Specialist nurse x2<br>30 mins Dietician x2	30 mins Consultant paediatrician x2<br>30 mins Specialist nurse x2<br>30 mins Dietician x2	30 mins Consultant paediatrician x2<br>30 mins Specialist nurse x2<br>30 mins Dietician x2
Costs	£360	£480	£480	£360	£240	£240	£240

Bloods	Plasma quantitative amino acids Total homocysteine	Plasma quantitative amino acids x27 Total homocysteine x27 B12 Folate	Plasma quantitative amino acids x6 Total homocysteine x6 B12	Plasma quantitative amino acids x4 Total homocysteine x4 B12	Plasma quantitative amino acids x4 Total homocysteine x4 B12	Plasma quantitative amino acids x4 Total homocysteine x4 B12	Plasma quantitative amino acids x4 Total homocysteine x4 B12
Costs	£115	£2,990	£702	£472	£472	£472	£472
Diet		HCU anamix to give 75ml per day Pyridoxine 50mg per day Folate acid 5mg per day30 mins Dietician x14 (additional apps)	HCU gel, 1 24g sachet per day, (10g)	HCU gel, 1 24g sachet per day, (10g)	HCU gel, 1 24g sachet per day, (10g)	HCU cooler, 1 130ml sachet per day, (15g)	HCU express 20, 1 34g sachet per day , (20g)
Supplements costs		£2,354	£2,334	£2,334	£2,334	£3,478	£4,778
Other		Baseline ophthalmology	Ophthalmology	Ophthalmology	Ophthalmology Psychometric assessment (at 4)	Ophthalmology Psychometric assessment (at 10)	Dexa Scan (yearly from 16)
Other		£498	£109	£250	£250	£250	£179
Total costs	£475	£6,322	£3,625	£3,416	£3,296	£4,440	£5,669

mins – minutes, HCU - homocystinuria

Table S2c – IVA costs – High risk

	Diagnosis	0-1	1-5	6-7	7-15	16+
Appointments	90 mins Consultant paediatrician<br>90 mins Specialist nurse<br>90 mins Dietician<br>+ 30 mins each for confirmation appointment	30 mins Consultant paediatrician x3<br>30 mins Specialist nurse x3<br>30 mins Dietician x3<br>30 mins Dietician x3 (additional apps)	30 mins Consultant paediatrician x4<br>30 mins Specialist nurse x4<br>30 mins Dietician x4	30 mins Consultant paediatrician x2<br>30 mins Specialist nurse x2<br>30 mins Dietician x2	30 mins Consultant paediatrician x2<br>30 mins Specialist nurse x2<br>30 mins Dietician x2	30 mins Consultant
Costs	£600	£360	£480	£240	£240	£79
Bloods	Plasma quantitative amino acids<br>Urinary/organic acids<br>Blood plasma acyl carnatine<br>DNA IVA	FBC x3<br>Ca, Pi, LFT x3<br>Plasma quantitative amino acids x3<br>Free carnitines & acyl carnitine x3<br>Micronutrients	FBC x4<br>Ca, Pi, LFT x4<br>Plasma quantitative amino acids x4<br>Free carnitines & acyl carnitine x4<br>Micronutrients	FBC x2<br>Ca, Pi, LFT x2<br>Plasma quantitative amino acids x2<br>Free carnitines & acyl carnitine x2<br>Micronutrients	FBC x2<br>Ca, Pi, LFT x2<br>Plasma quantitative amino acids x2<br>Free carnitines & acyl carnitine x2<br>Micronutrients	FBC<br>Ca, Pi, LFT<br>Plasma quantitative amino acids<br>Free carnitines & acyl carnitine<br>Micronutrients
Costs	£456	£573	£776	£394	£394	£203
Diet	Carnitine 100mg/kg/day	Carnitine 100mg/kg/day<br>Energivit 30ml/kg/day<br>IVA anamix 30ml/kg/day	Carnitine 100mg/kg/day<br>Paediatric seravit 17g per day (increases 1-2g per day each year)	Carnitine 100mg/kg/day<br>Paediatric seravit 24g per day (increases 1-2g per day each year)	Carnitine 100mg/kg/day<br>Paediatric seravit 27g per day (increases 1-2g per day each year)	Carnitine 50mg -100mg/kg/day

			Nutrini 200ml one per day	Nutrini 200ml one per day	Paediasure 500ml one per day	
Supplements costs	£21	£2,460	£3,043 (average 5 years)	£4,058	£6,707 (average 9 years)	£3,885
Other		£51				
Total costs	£1077	£3,444	£4,299	£4,692	£7,341	£4,167

Mins – minutes, FBC – full blood count, ca – calcium, Pi – perfusion index, LFT – liver function tests, g – gram, kg - kilogram

Table S2d – IVA costs – Low risk

	Diagnosis	0-1	1-5	6-15	16+
Appointments	90 mins Consultant paediatrician<br>90 mins Specialist nurse<br>90 mins Dietician<br>+ 30 mins each for confirmation appointment	30 mins Consultant paediatrician x1.5<br>30 mins Specialist nurse x1.5<br>30 mins Dietician x1.5<br>30 mins Dietician x1.5(additional apps)	30 mins Consultant paediatrician x2<br>30 mins Specialist nurse x2<br>30 mins Dietician x2	30 mins Consultant paediatrician x1<br>30 mins Specialist nurse x1<br>30 mins Dietician x1	30 mins Consultant x1
Costs	£600	£180	£240	£120	£79
Bloods	Plasma quantitative amino acids<br>Urinary/organic acids<br>Blood plasma acyl carnatine<br>DNA IVA	FBC x1.5<br>Ca, Pi, LFT x1.5<br>Plasma quantitative amino acids x1.5<br>Free carnitines & acyl carnitine x1.5<br>Micronutrients	FBC x2<br>Ca, Pi, LFT x2<br>Plasma quantitative amino acids x2<br>Free carnitines & acyl carnitine x2<br>Micronutrients	FBC x1<br>Ca, Pi, LFT x1<br>Plasma quantitative amino acids x1<br>Free carnitines & acyl carnitine x1<br>Micronutrients	FBC x1<br>Ca, Pi, LFT x1<br>Plasma quantitative amino acids x1<br>Free carnitines & acyl carnitine<br>Micronutrientsx1
Costs	£456	£287	£388	£197	£203

Diet	£21				
Total costs	£1,077	£467	£628	£317	£282

Mins – minutes, FBC – full blood count, ca – calcium, Pi – perfusion index, LFT – liver function tests

Table S2e – LCHADD costs

	Diagnosis	0-1	1-18
Appointments	90 mins Consultant paediatrician<br>90 mins Specialist nurse<br>90 mins Dietician<br>+ 60 mins each for confirmation appointment	30 mins Consultant paediatrician x4<br>30 mins Specialist nurse x4<br>30 mins Dietician x4	30 mins Consultant paediatrician x4<br>30 mins Specialist nurse x4<br>30 mins Dietician x4
Costs	£360	£480	£480
Bloods	Blood acylcarnites<br>Urine Organic Acids<br>Genetic confirmation	Weekly plasma amino acids until 4 months – bi-weekly after	Bi-weekly plasma amino acids
Costs	£315	£416	£313
Diet		Lipistart 2g/kg/day	
Supplements costs		£254	
Total costs	£675	£1,150	£793

Mins – minutes, g – gram, kg - kilogram

Table S2f: MSUD costs

	Diagnosis	0-1	1-5	6-10	11-15	16+
Appointments	60 mins Consultant paediatrician<br>60 mins Specialist nurse<br>60 mins Dietician + 30 mins each for confirmation	30 mins Consultant paediatrician x4<br>30 mins Specialist nurse x4<br>30 mins Dietician x4	30 mins Consultant paediatrician x4<br>30 mins Specialist nurse x4<br>30 mins Dietician x4	30 mins Consultant paediatrician x2<br>30 mins Specialist nurse x2<br>30 mins Dietician x2	30 mins Consultant paediatrician x2<br>30 mins Specialist nurse x2<br>30 mins Dietician x2	30 mins Consultant
Costs	£360	£480	£480	£480	£480	£480
Bloods	Urinary/organic acids<br>Blood plasma AA<br>DNA MSUD	Weekly BCAA monitoring until 8 weeks<br>Bi-weekly BCAA thereafter	Bi-weekly BCAA monitoring	Bi-weekly BCAA monitoring	Bi-weekly BCAA monitoring	Bi-weekly BCAA monitoring
Costs	£115	£313	£313	£313	£313	£313
Diet		3g/kg/day MSUD Anamix<br>Isoleucine and valine supplements 300mg per day<br>Protein exchanges	3g/kg/day MSUD Gel<br>Isoleucine and valine supplements 300mg per day<br>Protein exchanges	3g/kg/day MSUD Gel<br>Isoleucine and valine supplements 300mg per day<br>Protein exchanges	3g/kg/day MSUD Gel<br>Isoleucine and valine supplements 300mg per day<br>Protein exchanges	3g/kg/day MSUD Gel<br>Isoleucine and valine supplements 300mg per day<br>Protein exchanges
Supplements costs		£8,318	£10,852 (average)	£12,502 (average)	£16,621 (average)	£16,772 (average)
Total costs	£475	£9,111	£11,654	£13,295	£17,414	£17,565

Mins- minutes, BCAA – branched chain amino acids, MSUD – maple syrup urine disease, g – gram, kg - kilogram

S3: Health and social care costs of crises and long-term outcomes

The costs associated with an encephalopathy crisis for GA1, MSUD, and LCHADD and preventative emergency admissions are shown in Table S3a. The length of stay was estimated from an expert panel and the cost per day and dialysis were taken from NHS Reference Costs [4]. The frequency of encephalopathy crises were estimated from Kolker et al [5] and an expert panel. The frequency of preventative hospital admissions, shown in Table S3b, were estimated from an expert panel.

Table S3a: Duration and costs of encephalopathy crises and episodes of intercurrent illness requiring preventive management

**Initial encephalopathy crisis**	Mean	Lower	Upper
Duration (days)	24	21	28
NICU/PICU cost	£1,117	£844	£1,307
Dialysis	£198	£167	£167
Dialysis sessions per week	3	3	3
Total	£28,845	£19,218	£38,606
**Second encephalopathy crisis**			
Duration (days)	4	2	7
NICU/PICU cost	£1,117	£844	£1,307
Dialysis	£198	£167	£167
Dialysis sessions per week	3	3	3
Total	£4,808	£1,830	£9,652
**Preventive emergency hospital admissions**			
Duration – Normal child (days)	3	2	4
NICU/PICU cost	£440	£347	£480
Total	£1,321	£695	£1,920
Duration – Dystonic child (days)	4	2	7
NICU/PICU cost	£795	£662	£927
Total	£3,181	£1,324	£6,486

NICU – Neonatal intensive care unit, PICU – Paediatric intensive care unit

Table S3b: Frequency and annual costs of episodes of intercurrent illness requiring preventive management.

Age (years)	Episode frequency			Preventive episodes requiring hospitalisation	
	Mid	Min	Max	Asymptomatic	With dystonia
1	4	2	6	£5,283	£3,391
2	2	1	4	£2,641	£6,362
3	2	1	4	£2,641	£6,362
4	2	1	4	£2,641	£6,362
5	2	1	4	£2,641	£6,362
6	2	1	4	£2,641	£6,362
6 - 12	2	1	4	£0	£6,362

For all conditions the costs associated with living with developmental delay or neurological impairment were estimated from the PSSRU Unit Costs of Health and Social Care 2012 using autism [2] as a proxy. The costs per year are shown in Table S3c

Table S3c: Health and social care costs associated with neurological impairment/developmental delay

Health state	Age			
	0-3	4-11	12-17	18+
Mild	£ 652	£ 23,265	£ 23,265	£9,009
Moderate	£ 1,353	£ 23,339	£ 38,221	£41,808
Severe	£ 1,406	£ 50,336	£ 82,432	£90,168
Mild/moderate	£652	£23,339	£38,221	£52,077

For HCU the incidence, type and timing of surgery for lens removal and retinal detachment in respect of the ocular problems of HCU are taken from Taylor et al [6]. The unit costs of surgery are taken from the NHS Reference Costs [4] and are estimated at £197.10 for correcting retinal detachment and £1440.43 for lens removal. Surgery is estimated to be required in 75% of symptomatically detected HCY and the costs are applied at the age of 9 years.

Figure S1: Cost-effectiveness planes

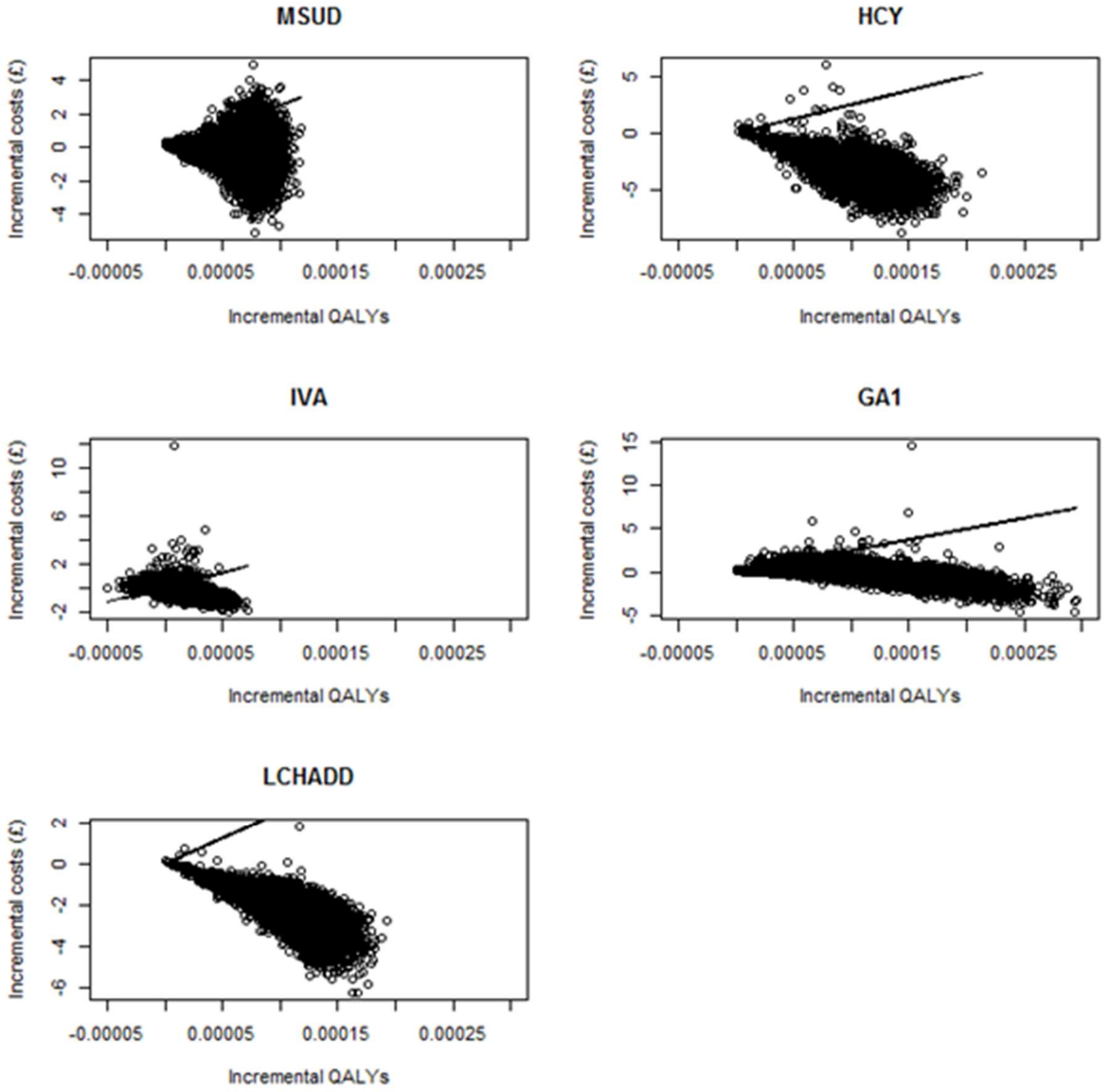

Each circle represents one model run. The diagonal line represents the cost-effectiveness threshold at £25,000 per quality adjusted life year (QALY). Each circle below the line represents a model run that is below the threshold of £25,000 per QALY and each circle above the line represents a model run that is a above the threshold.

Figure S2: QALYs in symptomatically and screen detected cases

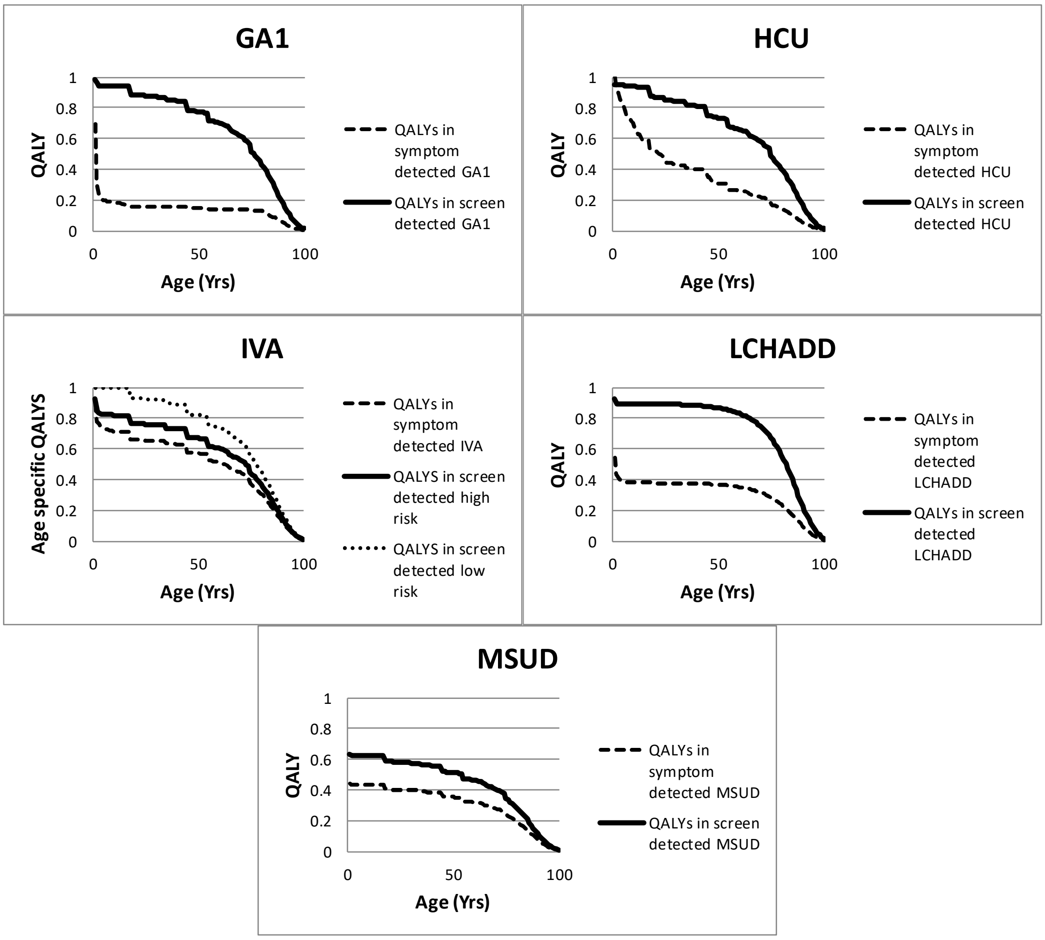

This figure shows the modelled QALY estimates against age for each of the five conditions.

References

1. Sheffield Children's Hospitals NHS Foundation Trust. Expanded Newborn Screening. Availabe online: http://www.expandedscreening.org/site/home/start.asp (accessed on 13th February 2012).
2. Curtis, L. *Unit Costs of Health and Social Care 2012*; University of Kent: Canterbury, 2012.
3. Paediatric Formulary Committee. *BNF for Children*; BMJ Group, Pharmacuetical Press, and RCPCH Publications: London, 2011-2012.
4. Department of Health. NHS Reference Costs 2011 to 2012. Availabe online: https://www.gov.uk/government/publications/nhs-reference-costs-financial-year-2011-to-2012 (accessed on 4th July 2012).
5. Kölker, S.; Garbade, S.F.; Boy, N.; Maier, E.M.; Meissner, T.; Mühlhausen, C.; Hennermann, J.B.; Lücke, T.; Häberle, J.; Baumkötter, J., et al. Decline of Acute Encephalopathic Crises in Children with Glutaryl-CoA Dehydrogenase Deficiency Identified by Newborn Screening in Germany. *Pediatric Research* **2007**, *62*, 357-363, doi:10.1203/PDR.0b013e318137a124.
6. Taylor, R.H.; Burke, J.; O'Keefe, M.; Beighi, B.; Naughton, E. Ophthalmic abnormalities in homocystinuria: the value of screening. *Eye (Lond)* **1998**, *12 (Pt 3a)*, 427-430, doi:10.1038/eye.1998.100.
